# The impact of the COVID-19 pandemic on routine HIV care and cervical cancer screening in North-Central Nigeria

**DOI:** 10.1186/s12905-023-02782-6

**Published:** 2023-11-30

**Authors:** Magdiel A. Habila, Mavis Obeng-Kusi, Maryam J. Ali, Francis A. Magaji, Iornum H. Shambe, Patrick H. Daru, Elizabeth T. Jacobs, Purnima Madhivanan, Atiene S. Sagay, Jonah Musa

**Affiliations:** 1 Mel and Enid, Department of Epidemiology and Biostatistics, Zuckerman College of Public Health, University of Arizona, Tucson, AZ USA; 2 Department of Health and Pharmaceutical Outcomes, R. Ken Coit College of Pharmacy, University of Arizona, Tucson, AZ USA; 3 Department of Obstetrics and Gynecology, University of Jos and Jos University Teaching Hospital, Jos, Plateau State Nigeria; 4 Mel and Enid, Department of Health Promotion Sciences, Zuckerman College of Public Health, University of Arizona, Tucson, AZ USA; 5 Public Health Research Institute of India, Mysore, India; 6 Department of Preventive Medicine, Division of Cancer Epidemiology and Prevention, Northwestern University Feinberg School of Medicine, Chicago, IL USA

**Keywords:** HIV management, HAART adherence, Pap smear, COVID-19, Cervical cancer screening

## Abstract

**Introduction:**

Cervical cancer is the fourth most diagnosed cancer among women globally, with much of the burden being carried by women in limited-resource settings often worsened by the high prevalence of HIV. Furthermore, the COVID-19 pandemic disrupted organized screening efforts and HIV management regimens worldwide, and the impact of these disruptions have not been examined in these settings. The purpose of this paper is to describe whether uptake of cervical cancer screening and HIV management changed before, during, and since the COVID-19 pandemic in North-Central Nigeria.

**Methods:**

Longitudinal healthcare administration data for women who obtained care between January 2018 and December 2021 were abstracted from the AIDS Prevention Initiative Nigeria (APIN) clinic at Jos University Teaching Hospital. Patient demographics, pap smear outcomes, and HIV management indicators such as viral load and treatment regimen were abstracted and assessed using descriptive and regression analyses. All analyses were conducted comparing two years prior to the COVID-19 pandemic, the four quarters in 2020, and the year following COVID-19 restrictions.

**Results:**

We included 2304 women in the study, most of whom were between 44 and 47 years of age, were married, and had completed secondary education. About 85% of women were treated with first line highly active retroviral therapy (HAART). Additionally, 84% of women screened using pap smear had normal results. The average age of women who sought care at APIN was significantly lower in Quarter 3, 2020 (*p* = 0.015) compared to the other periods examined in this study. Conversely, the average viral load for women who sought care during that period was significantly higher in adjusted models (*p* < 0.0001). Finally, we determined that the average viral load at each clinic visit was significantly associated with the period in which women sought care.

**Conclusions:**

Overall, we found that COVID-19 pandemic mitigation efforts significantly influenced women’s ability to obtain cervical cancer screening and routine HIV management at APIN clinic. This study buttresses the challenges in accessing routine and preventive care during the COVID-19 pandemic, especially in low-resource settings. Further research is needed to determine how these disruptions to care may influence long-term health in this and similar at-risk populations.

## Introduction

Cervical cancer is one of the most commonly- diagnosed cancers among women worldwide [1]. Recent global estimates suggest that the incidence of cervical cancer is 13.1 per 100,000 women, with a mortality rate of 6.9 per 100,000 in 2018 [1]. A significant proportion of cervical cancer incidence and mortality is among women living in low-and-middle-income countries (LMICs) [1, 2]. In Nigeria, the most populous country in sub-Saharan Africa, cervical cancer is the second most common cancer among women, with an incidence rate of 18.4 per 100,000 women and mortality rate of 13.2 per 100,000 women in 2020 [3]. In Nigeria, as with other LMICs, research suggests that the incidence and mortality due to cervical cancer has increased over time due to lack of access to primary prevention in the form of HPV vaccination, and limited access to and uptake of cervical cancer screening including visual inspection with acetic acid, pap smear, and/or HPV DNA testing [1, 4].

HPV DNA testing has become the global standard of care for cervical cancer screening in high-income countries (HIC) for its superiority to cytology-based approaches to screening [4]. However, due to the economic and health system challenges this approach would present in low-resource settings, the World Health Organization (WHO) recommendations still support the use of pap smear as the gold standard for cervical cancer screening in low-resource settings [4, 5]. The implementation of pap smear has challenges in LMICs because of the limited number of health centers that have cytopathologist and other resources for pap cytology testing, interpretation, and reporting of results to women after a completed screening [2, 6, 7]. Though some of these challenges have been addressed through the use of alternative screening options such as visual inspection with acetic acid or Lugol’s iodine in rural settings and primary healthcare centers in many LMIC communities, many behavioral, social, and cultural barriers persist that hinder women from accessing screening [3, 7, 8].

Furthermore, many LMICs have a high prevalence of HIV, an important risk factor for cervical cancer [1]. In 2022, there were an estimated 39 million people living with HIV globally, including 1.3 million new cases of HIV infection, with 46% of those new cases being among women. Estimates also indicate that of the 39 million people, approximately 25 million people are living in Africa [9, 10]. Nigeria has the second highest number of people living with HIV in the world with an estimated 2 million people living with HIV [11]. In spite of this high disease prevalence, awareness about HIV ranges between 9% and 89% depending on geographical region, age, income, and level of education among other factors [12–14]. UNAIDS estimate that in 2020, 90% of people living with HIV in Nigeria knew their status and 86% of them are receiving antiretrovial therapies (ARTs) [15]. Knowledge about cervical cancer screening and uptake of screening where available, on the other hand, has remained poor in the general population and among women living with HIV prior to the COVID-19 pandemic [3, 16, 17]. Poor uptake of screening among women living with HIV is cause for concern since their risk of cervical cancer is 6-times that of women uninfected by HIV [18]. This suggests that though steps have been taken at healthcare institutions to address some of the barriers to screening uptake that women living with HIV face to accessing screening, many of those barriers persist.

The COVID-19 pandemic brought notable disruptions to the management of chronic diseases globally. The cancer care continuum, and prevention and control programs in the United States and globally experienced disruptions due to nation-wide lockdowns imposed to reduce the spread of the virus and were among the most effective public health measures instituted by governments worldwide [2, 6, 19]. While these lockdowns, work-from-home practices and other efforts were highly effective for those living in high-income countries (HICs), the impact of this practice on cancer prevention and control in the US is not fully understood. A recent study found that cervical cancer screening in the United States dropped by 94% following COVID-19 lockdowns and remained below 35% even after restrictions were lifted [6]. Researchers also found that patients missed important cancer prevention benchmarks such as diagnostic procedures and chemotherapy appointments, to prevent exposure to COVID-19 [19, 20]. The impact that the COVID-19 pandemic had on access to routine HIV care globally also remains unclear, though researchers have posited that since these two diseases have disproportionately affected underserved communities, action is needed in order to prevent exacerbating pre-existing disparities in outcomes associated with HIV infection and the COVID-19 pandemic [21].

In low-resource settings, studies evaluating the impact of COVID-19 pandemic and the ensuing lockdowns on the cancer care continuum are scarce, with one study reporting changes in cancer treatment but not screening [22]. This gap in research is partially due to the lack of representative population-based cancer registries and a limited availability to describe trends in cervical cancer screening [23]. Though studies have been conducted to examine the impact of the pandemic on routine HIV care in LMICs, findings from these studies vary significantly by population and health system [24, 25]. The observed heterogeneity in the literature on the impact of the COVID-19 pandemic serve as a reminders that communities in LMICs vary meaningfully from each other in and that there is a need to understand how community-level HIV and cervical cancer burden interact with the health system and policy implications of COVID-19 pandemic lockdowns so that healthcare providers in each community are equipped with knowledge of barriers to care that their patients face in emergency situations.

In Nigeria, the lockdowns were instituted nation-wide, limiting access to food, transportation, houses of faith, and work, and the lack of adequate telecommunication infrastructure limited access to tele-work opportunities. For healthcare professionals who needed to physically be present at work, limited availability of personal protective equipment (PPE) reduced the services healthcare providers could offer to patients, created additional barriers in access to healthcare facilities including routine HIV care, access to highly active antiretroviral therapies (HAART), and cervical cancer screening. The goal of the present analysis, therefore, is to assess the use of cervical cancer screening and routine HIV management before, during, and after the lockdowns in response to the COVID-19 pandemic at a tertiary healthcare facility in North-Central Nigeria.

## Methods

We conducted a secondary analysis using data from the AIDS Prevention Initiative Nigeria (APIN) clinic at Jos University Teaching Hospital between January 2018 and December 2021. Various services that cater specifically to people living with HIV are available at no direct cost to the patients receiving care within APIN including HIV counseling, laboratory services including viral load and CD4 count monitoring, tuberculosis (TB) screening and treatment, pharmacy drug pickups, sexually transmitted infection (STI) screening, and cervical cancer screening offered through Operation Stop Cervical Cancer (OSCC). OSCC began opportunistic screening and treatment for cervical precancer in 2006 through support provided by Exxon Mobil, Texas, USA, through the African Organization for Research and Training in Cancer (AORTIC) [26]. OSCC also provides access to contraceptive services upon request.

All the departments within APIN maintain detailed physical and electronic medical records for all patients who are enrolled in routine care at APIN including demographic characteristics (age, religion, state of residence, marital status), clinical factors pertaining to adherence to HIV treatment (viral load, CD4 counts, missed appointments), risk factors (previous sexually transmitted infections, HIV status, parity, oral contraceptive use), and clinical outcomes (outcomes of pap smear) of women who were screened at the clinic, and other relevant outcomes. Women received all necessary HIV care services at APIN during a routine clinic day and were referred for cervical cancer screening by a healthcare provider or self-referral. We included data for all women living with HIV who were screened for cervical cancer between January 2018 and December 2021. As our primary outcomes of interest were adherence to HIV care and cervical cancer screening access, men and people who were not infected with HIV were excluded from these analyses. Additionally, women with HIV viral load above 100,000 copies per mL of blood were excluded from the analyses as this viral load was greater than two standard deviations above the mean.

A de-identified dataset was created of records from women living with HIV who were screened for cervical cancer between January 2018 and December 2021. The database included demographics, clinical, HIV management, history of STI, and pap smear outcomes. The primary analyses were conducted on all women meeting the inclusion criteria of these analyses. Data were categorized as follows: women screened in 2018 and 2019 (classified as the pre-COVID pandemic period), women screened between January and March 2020 (Quarter 1 2020), women screened between April and June 2020 (Quarter 2 2020), women screened between July and September 2020 (Quarter 3 2020), women screened between October and December 2020 (Quarter 4 2020), and finally women screened during 2021 (post-COVID pandemic period). Descriptive analyses were conducted using the six groups described above; means and standard deviations were used for continuous variables, and analyses of variance for categorical variables. Statistical significance was set at *p* ≤ 0.05 for descriptive analyses. Data for these analyses were aggregated and analyzed in 2020 using STATA v.16 [Stata Corp, College Station, TX].

APIN maintains longitudinal records of all patients using a unique patient identifier, that we were able to assess the impact of COVID-19 pandemic mitigation efforts on patient access to routine HIV management and cervical cancer screening cross-sectionally and longitudinally. We used the cross-sectional approach to identify the number of women who obtained care (pap smear and HIV management) at APIN before, during, and after COVID-19 pandemic mitigation efforts. All eligible women were included in these analyses. However, to obtain a cross-sectional sample of these data, we identified the “first” visit for each woman who obtained care between 2018 and 2021 as indicated by the earliest date at which each woman was seen at APIN. A dataset was created which included demographic and clinical data for each woman on the earliest date she obtained care between 2018 and 2021. The primary analysis assessed two questions; first, how many women obtained routine care for HIV and cervical cancer screening before, during, and after COVID-19 mitigation efforts were instituted, and second, to describe how demographic factors such as age, level of education, marital status of women, and pap smear use during these periods changed. The analysis results displayed in Tables 1, 2 and 3 were conducted using the cross-sectional dataset.

**Table 1:** Descriptive statistics of women who obtained care at AIDS Prevention Initiative of Nigeria (APIN) between 2018 and 2021 (N = 2,304)

	2018–2019 Pre-COVID Restrictions	2020 Quarter 1 January – March	2020 Quarter 2 April – June	2020 Quarter 3 July – September	2020 Quarter 4 October – December	2021 Post-COVID Restrictions
Mean ± Standard Deviation						
**Age (in years)**	46.7 ± 7.9	46.7 ± 8.9	45.3 ± 8.3	44.3 ± 8.1	47.3 ± 7.7	47.1 ± 8.7
**Viral Load (copies per mL blood)** ^**a**^	107.6 + 1203.0	48.4 + 197.2	354.1 + 1765.9	2207.4 + 11989.5	188.1 + 1690.4	488.3 + 5134.4
	N (%)					
**Education**						
No Formal Education	131 (37.2)	22 (6.3)	8 (2.3)	10 (2.8)	48 (13.6)	133 (37.8)
Primary (Grade 1–8)	218 (41.8)	36 (7.0)	4 (0.8)	13 (2.5)	76 (14.6)	175 (33.5)
Secondary (Grade 9–12)	300 (40.8)	45 (6.1)	8 (1.1)	33 (4.5)	85 (11.6)	264 (35.9)
Tertiary (Higher Education)	253 (37.6)	37 (5.5)	8 (1.2)	22 (3.3)	99 (14.7)	254 (37.7)
**Marital Status**						
Single	195 (39.2)	25 (5.0)	5 (1.0)	18 (3.6)	67 (13.5)	188 (37.8)
Separated	20 (34.5)	8 (13.8)	0 (0)	3 (5.2)	9 (15.5)	18 (31.0)
Married	462 (39.6)	81 (7.0)	16 (1.4)	42 (3.6)	154 (13.2)	411 (35.3)
Divorced	43 (36.4)	9 (7.6)	3 (2.5)	2 (1.7)	15 (12.7)	46 (39.0)
Widowed	182 (41.2)	17 (3.9)	4 (0.9)	13 (2.9)	63 (14.3)	163 (36.9)
**Antiretroviral Regimen Line**						
First Line	773 (39.6)	117 (6.0)	22 (1.1)	65 (3.3)	277 (14.2)	699 (35.8)
Second Line	129 (39.3)	23 (7.0)	6 (1.8)	13 (4.0)	31 (9.5)	126 (38.4)
Third Line ^b^						
**Pap Smear Outcome**						
Normal	755 (36.2)	130 (6.2)	30 (1.4)	91 (4.4)	319 (15.3)	762 (36.5)
Low-grade Abnormalities (ASC-US, LSIL, AC-US)	126 (41.6)	24 (7.9)	0	5 (1.7)	68 (22.4)	80 (26.4)
High-grade Abnormalities (AGUS, HSIL, HSIL, suspect invasion)	9 (10.5)	3 (3.5)	0	0	17 (19.8)	57 (66.3)

^a^ HIV Viral load recorded above 100,000 copies per mL of blood were excluded from all analyses

^b^ Insufficient sample size to display data

**Table 2:** Linear regression model illustrating mean difference in age of women who sought care at APIN by COVID period

	N	M ± SE	Unadjusted ß (95% CI)	p-value
**Pre-COVID (2018–2019)**	902	46.7 + 0.26	Reference	
2020 Quarter 1 (January – March)	140	46.7 ± 0.74	0.02 (-1.43, 1.49)	0.97
2020 Quarter 2 (April – June)	28	45.3 ± 1.58	-1.45 (-4.55, 1.64)	0.35
2020 Quarter 3 (July – September)	78	44.3 ± 0.97	-2.37 (-4.28, -0.46)	**0.015**
2020 Quarter 4 (October – December)	208	47.3 ± 0.54	0.63 (-0.43, 1.69)	0.24
2021 Post COVID Restrictions	826	47.1 ± 0.39	0.37(-0.40, 1.14)	0.35

**Table 3:** Linear regression model illustrating mean difference in HIV viral load for women who sought care at APIN by COVID period

	N	M ± SE	Unadjusted ß (95% CI)	p-value	Adjusted ß ^a^ (95% CI)	p-value
**Pre-COVID Restrictions**	891	107.3 ± 40.3	Reference			
2020 Quarter 1 (January – March)	138	48.2 ± 359.5	-59.2 (-764.1, 645.7)	0.86	-58.2 (-762.1, 645.5)	0.87
2020 Quarter 2 (April – June)	27	354.1 ± 767.6	246.4 (-1258.9, 1751.7)	0.74	195.4 (-1307.9, 1696.6)	0.79
2020 Quarter 3 (July – September)	77	2207.4 ± 466.8	2099.8 (1184.5, 3015.1)	**< 0.0001**	2029.8 (1114.8, 2944.9)	**< 0.0001**
2020 Quarter 4 (October – December)	303	188.1 ± 261.3	80.4 (-432.0, 593.0)	0.75	95.8 (-415.9, 607.6)	0.71
2021 Post COVID Restrictions	819	488.3 ± 190.2	380.7 (7.6, 753.7)	**0.04**	390.2 (17.72, 762.7)	**0.04**

^a^ Adjusted for current age

Previous literature suggests that people living with HIV in this region may have increased use of healthcare services. To account for this, we also examined these data longitudinally to assess whether the relevant patient outcomes changed significantly among women who were able to obtain care multiple times between 2018 and 2021. To achieve this, we compiled a second dataset consisting of data from every visit for all women who obtained care between 2018 and 2021. Using linear regression models, we sought to describe how, if at all, COVID-19 pandemic mitigation efforts impacted patient adherence to HIV management regimens as measured by changes in HIV viral load across the periods previously described. To account for potential confounding between more severe illness and greater number of clinic visits, we stratified these analyses by number of clinic visits, enabling us to identify changes in HIV viral load over the periods in question, stratified by frequency of clinic visits which serves as a proxy for disease severity. The analysis results displayed in Table 4 were conducted using the longitudinal dataset. In all analyses, recorded viral loads over 100,000 copies/mL of blood were excluded from the analyses as outliers which significantly influenced analysis results.

**Table 4:** Linear regression models comparing mean differences in HIV viral load by COVID period and number of visits between 2018 and 2021

	N	M ± SE	Unadjusted ß (95% CI)	p-value	Adjusted ß ^a^ (95% CI)	p-value
**Women who had 1 Visit**	1747					
**Pre-COVID Restrictions**	517	65.7 ± 22.4	Reference			
2020 Quarter 1 (January – March)	98	55.9 ± 22.8	-9.82 (-861.7, 842.0)	0.98	-15.0 (-865.6, 835.6)	0.97
2020 Quarter 2 (April – June)	23	412.4 ± 399.0	346.7 (-1301.0, 1994.3)	0.68	313.4 (-1331.3, 1959.0)	0.70
2020 Quarter 3 (July – September)	68	2498.3 ± 1545.0	2432.5 (1435.1, 3429.9)	**< 0.0001**	2375 (1379.0, 3372.7)	**< 0.0001**
2020 Quarter 4 (October – December)	281	205.2 ± 104.7	139.5 (-433.4, 712.6)	0.63	168 (-403.9, 741.6)	0.56
2021 Post COVID Restrictions	760	418.1 ± 161.8	352.3 (-88.5, 793.1)	0.11	389.9 (-51.3, 831.0)	0.08
**Women who had 2 Visits**	456					
**Pre-COVID Restrictions**	240	225.5 ± 140.9	Reference			
2020 Quarter 1 (January – March)	60	24.5 ± 7.7	-201.0 (-1340.4, 938.4)	0.72	-220.2 (-1353.9, 913.5)	0.70
2020 Quarter 2 (April – June)	8	409.0 ± 362.6	183.5 (-2653.5, 3020.6)	0.89	265.6 (-2557.3, 3088.6)	0.85
2020 Quarter 3 (July – September)	28	32.5 ± 21.7	-192.9 (-1769.4, 1383.4)	0.81	-90.6 (-1661.2, 1479.9)	0.91
2020 Quarter 4 (October – December)	104	13.2 ± 2.0	-212.2 (-1138.9, 714.5)	0.65	-140.1 (-1063.9, 783.7)	0.76
2021 Post COVID Restrictions	16	5088.5 ± 5046.9	4863.0 (2824.8, 6901.3)	**< 0.0001**	4883.9 (2855.6, 6912.2)	**< 0.0001**
**Women who had 3 Visits**	64					
**Pre-COVID Restrictions**	9	33.3 ± 22.0	Reference			
2020 Quarter 1 (January – March)	18	15.9 ± 3.1	-17.4 (-162.4, 127.6)	0.81	-20.7 (-169.3, 127.9)	0.78
2020 Quarter 2 (April – June)	4	18.8 ± 8.8	-14.5 (-228.0, 198.9)	0.89	-22.4 (-242.8, 198.1)	0.84
2020 Quarter 3 (July – September)	6	227.7 ± 209.9	194.3 (7.1, 381.5)	0.42	189.2 (-3.5, 381.8)	0.05
2020 Quarter 4 (October – December)	27	43.8 ± 26.6	10.4 (-126.3, 147.2)	0.87	12.77 (-126.7, 152.2)	0.85

^a^ Adjusted for age and level of education

## Results

Table 1 displays the demographic and clinical characteristics of the 2304 women included in these analyses by the periods described above. On average, women were between 44 and 47 years old, married, and had completed at least secondary school education. Most women included in this study were being treated with first-line antiretroviral therapy (85.5%), with average recorded viral load ranging from 107.6 to 2207.4 copies per mL of blood, with the lowest average viral load observed in women obtaining care before the COVID-19 pandemic and the highest average viral loads being observed in women obtaining care between July and September 2020. We observed a significant decrease in the number of women who were screened for cervical cancer between April and September 2020. The proportions of normal pap smears between the period prior to the COVID-19 pandemic restrictions and the period after those restrictions were similar (36.2% vs. 36.5%). However, the proportion of high-grade abnormalities detected in the period after the COVID-19 pandemic restrictions was significantly higher than the period prior to COVID-19 restrictions (66.3% vs. 10.5%).

We found that the number of women who were able to obtain routine care for HIV decreased in 2020, with the fewest number of women obtaining care between April and September 2020 (Table 1). A similar trend was observed pertaining to the number of women who were able to receive pap smear, where screening uptake decreased in 2020, with the most notable decrease being between April and September of 2020 (Table 1).

Level of education and marital status were not related to obtaining care across the six periods assessed in this study (data not shown), but age was associated with obtaining care. Specifically, the average age for women who were seen for routine care for HIV between January and September 2020 decreased significantly compared to the average age of women who obtained care before the COVID-19 pandemic, as is shown in Table 2. Compared to women seeking care at APIN before the COVID-19 pandemic restrictions, women who received care between July and September 2020 were on average two years younger than women who were seen at APIN before the pandemic (*p* = 0.01, see Table 2).

Concerning changes in patient HIV viral load, we identified that compared to the average viral load of women who obtained care at APIN before the COVID-19 pandemic, women who obtained care between July and September 2020 and in 2021 had significantly higher average viral loads, with 107.3 vs. 2207.4 copies/mL of blood (*p* < 0.0001) and 107.3 vs. 488.3 copies/mL of blood (*p* = 0.04), respectively, as displayed in Table 3.

As shown in Table 4, we classified women as having one or more visits to APIN before COVID-19 pandemic mitigation efforts were put in place, in each quarter of 2020, and in 2021. We identified that 76.7% of women were seen at APIN between 2018 and 2021. Among these women who had one visit between 2018 and 2021, we observed an increasing trend in average viral load with the peak detected among women who were treated between July and September 2020. These patients had significantly higher average viral load compared to women who obtained care before the COVID-19 pandemic, with 65.7 vs. 2498.3 copies/mL of blood for each group, respectively (*p* < 0.0001). We observed a second peak in the average viral load among women who obtained care after COVID-19 pandemic restrictions, but that increase was not statistically significant (*p* = 0.11).

Finally, we observed decreased average viral load among the women who obtained care twice between January and March 2020 (*p* = 0.72) followed by increased average viral load for the women who obtained care twice in the following quarter (*p* = 0.89) compared to women who obtained care before the COVID-19 pandemic. This increase was followed by two quarters in which the average viral load decreased, though both changes were not significant compared to women who obtained care before the COVID-19 pandemic. However, among women who obtained care twice in 2021, we observed a significant increase in average viral load compared to women who obtained care before the COVID-19 pandemic, with the average viral load of 5088 copies/mL of blood (< 0.0001). The average viral load among women who obtained care three times within the study period did not vary significantly in 2020 or in 2021 compared to the pre-COVID pandemic period. However, we observed increased average viral load for women who obtained care three times between July and September (*p* = 0.42). We observed a similar trend among women who obtained care four times during the study period, where trends suggest a non-significant increase in average viral load compared to the period before the COVID-19 pandemic, but these women remained virally suppressed regardless of the period in which they sought care (data not shown).

## Discussion

Overall, we found that COVID-19 pandemic lockdowns had a significant impact on women’s ability to obtain routine cervical cancer screening and HIV management at APIN in Jos University Teaching Hospital. On average, younger women received care between April and September 2020, the periods immediately following COVID-19 pandemic lockdowns, as compared to the period before lockdowns began. We have also shown that cervical cancer screening uptake decreased significantly during these periods. Finally, we have shown that on average, patient viral load at clinic visits increased between April and September 2020 and then again in 2021, with the greatest increases in average viral load being observed among women who obtained care once between July and September, and women who obtained care twice in 2021.

As mentioned above, women who sought care in the periods immediately following the COVID-19 pandemic restrictions were on average younger than women who sought care before the COVID-19 pandemic restrictions. One possible explanation for this finding is that much of the early public health messaging emphasized that older individuals were at increased risk for COVID-19 infection. As such it is possible that younger women perceived that their risk for infection was lower and were more likely to seek routine care. One study conducted to assess risk perception in ten countries found that perceived lower risk of COVID-19 infection influenced their active engagement in local COVID-19 pandemic mitigation efforts [27]. To our knowledge, this is the first study conducted in a low resource setting that shows trends similar to those discussed in HICs.

Next, we learned that changes in patient viral load in our study sample depended on the period at which viral load was measured. More specifically, patients who had the first viral load assessed in the pre-COVID period had on average, lower viral load for the remainder of the study period while patients who had the first viral load assessed between July and September 2020 on average had higher viral loads for the remainder of the study period. While the literature around this finding in this setting is sparse, this finding seems to point to the impacts of delays in accessing HIV care because of COVID-19 pandemic lockdowns. In Nigeria, as in many parts of the world in March 2020, nationwide lockdowns were instituted and enforced to minimize the spread of COVID-19. However, in Nigeria, unlike most places in the world, this brief lockdown period was followed by a period in which the initial restrictions were scaled back to allow people to purchase food and attend to their businesses. This period also allowed patients to go to healthcare centers to obtain necessary care. To be in alignment with WHO requirements of social distancing, the APIN clinic significantly limited the number of patients who could be seen on the days when it was open, per local government regulations.

These institutional, national, and global COVID-19 pandemic mitigation policies may be a significant contributing factor to delays in patient care that resulted in the increased average viral load observed in this study. Studies examining the influence of the COVID-19 pandemic on HIV care in northeastern Nigeria, Ethiopia, and South Africa observed similar trends, providing supporting evidence to suggest that lockdown efforts negatively impacted initiation of and adherence to HIV care [24, 25, 28]. Interestingly, the study from South Africa, which examined HIV care at public and private healthcare centers, showed that patients seeking care at public healthcare centers may have experienced greater disruptions to care than those seeking care at private healthcare centers [24]. This observed interruption in accessing ARTs is estimated to be a significant contributor to HIV-related deaths in high burden areas [29]. Additionally, the increase in viral load among women with HIV has implications in advancing cervical carcinogenesis and development of invasive cervical cancer [17].

Furthermore, we found that the number of clinic visits, and the period in which those visits fell also significantly influenced patient viral load. It is expected that increased access to care, as measured by number of clinic visits within the study period, would lead to better HIV management outcomes using viral load as a surrogate marker. However, our study findings highlight that the benefit of increased access to care was attenuated by the COVID-19 pandemic mitigation efforts, with various COVID-19 periods showcasing statistically and clinically significant higher viral loads among patients who were able to obtain care more than once within that period. This finding is difficult to explain given how quickly policies and guidelines regarding COVID-19 pandemic mitigation changed in the months following global lockdowns in March 2020. However, one possible explanation is that delays in accessing ARTs early in the pandemic period led to drug resistance in some HIV treatment regimens, which is a consequence observed in other contexts, leading to an increased need for follow-up care that we observed [28, 30]. Regardless, this finding highlights a gap in our understanding of barriers within health systems and sociopolitical landscapes that influence patient access to care, further exacerbated by the COVID-19 pandemic. The literature examining interactions between COVID-19 infection and HIV infection and progression are inconclusive, with some studies failing to demonstrate the HIV infection increases susceptibility to COVID-19 and others illustrating the opposite [31, 32]. Further research is needed to examine whether co-infection with COVID-19 and HIV would influence HIV viral load.

### Strengths and limitations

This study has many notable strengths. First, the data collected during healthcare administration at APIN had very limited missing data, enabling us to conduct analyses cross-sectionally and longitudinally to compare patient outcomes before and after the COVID-19 pandemic. Additionally, this study adds to existing literature that illustrates specific interruptions to HIV care and cervical cancer screening due to the COVID-19 pandemic. However, the limitation on external validity to populations outside the study setting is duly acknowledged. All patients represented in this database are women living with HIV, thus the interpretations of these findings are limited to this population. However, we believe that these findings maybe applicable to similar low-resource settings that serve people living with HIV.

## Conclusions

The novelty of the COVID-19 pandemic and the variations in the policy measures and recommendations that were taken to mitigate the spread of SARS-CoV-2 potentially posed additional barriers to HIV care and cervical cancer screening uptake in North-Central Nigeria. This added difficulty in obtaining care may have had dire consequences in Nigeria because of the high prevalence of HIV infection, and on UNAIDS 95-95-95 targets for minimizing the burden of HIV globally [33]. Additionally, HIV infection presents an important risk factor for cervical cancer and requires consistent monitoring and management to minimize the progression and mortality due to cervical cancer. These findings contribute to the literature concerning the effect that the COVID-19 pandemic had on access to routine care in low-resource settings, particularly in Nigeria. We have illustrated here that uptake of pap smear reduced significantly after April 2020 and did not return to baseline levels even in 2021, post-COVID restrictions. We have also illustrated that uptake of routine HIV care also decreased significantly in April 2020, resulting in increased average viral load for women who obtained care from July 2020 and onward compared to baseline. These findings highlight the impact of disruptions to routine HIV care and cervical cancer screening, enabling healthcare providers to be better equipped to care for women living with HIV in future emergency situations. Future research is needed to explore how the delays in access to HIV management may have impacted cervical cancer progression among women in this and similar settings.

## Data Availability

The datasets for this study belong to the Operation Stop Cervical Cancer Unit of the Jos University Teaching Hospital (JUTH) and could be made available on reasonable request to corresponding author.

## References

[1] Arbyn M, Weiderpass E, Bruni L, de Sanjosé S, Saraiya M, Ferlay J (2020). Estimates of incidence and mortality of Cervical cancer in 2018: a worldwide analysis. The Lancet Global Health.

[2] Carneiro MM. Cervical cancer elimination: rising up to the challenge. Taylor & Francis; 2021. pp. 617–8. 10.1080/03630242.2021.1959843 34365917

[3] Yimer NB, Mohammed MA, Solomon K, Tadese M, Grutzmacher S, Meikena HK (2021). Cervical cancer screening uptake in Sub-saharan Africa: a systematic review and meta-analysis. Public Health.

[4] Organization WH. Global strategy towards the elimination of Cervical cancer as a public health problem. World Health Organization; 2020.

[5] Gultekin M, Ramirez PT, Broutet N, Hutubessy R (2020). World Health Organization call for action to eliminate Cervical cancer globally. Int J Gynecol Cancer.

[6] Wentzensen N, Clarke MA, Perkins RB (2021). Impact of COVID-19 on Cervical cancer screening: challenges and opportunities to improving resilience and reduce disparities. Prev Med.

[7] Chigbu CO, Onyebuchi AK, Ajah LO, Onwudiwe EN (2013). Motivations and preferences of rural Nigerian women undergoing Cervical cancer screening via visual inspection with acetic acid. Int J Gynecol Obstet.

[8] Sankaranarayanan R, Anorlu R, Sangwa-Lugoma G, Denny LA (2013). Infrastructure requirements for human papillomavirus vaccination and Cervical Cancer screening in Sub-saharan Africa. Vaccine.

[9] GHO. Number of people (all ages) living with HIV - Estimates by WHO region [Available from: https://apps.who.int/gho/data/node.main.620?lang=en.

[10] UNAIDS, Global HIV. & AIDS statistics — 2022 fact sheet [Available from: https://www.unaids.org/en/resources/fact-sheet.

[11] Onovo AA, Adeyemi A, Onime D, Kalnoky M, Kagniniwa B, Dessie M et al. Estimation of HIV prevalence and burden in Nigeria: a bayesian predictive modelling study. EClinicalMedicine. 2023;62. 10.1016/j.eclinm.2023.102098 PMC10393599 37538543

[12] Ukaegbu E, Alibekova R, Ali S, Crape B, Issanov A (2022). Trends of HIV/AIDS knowledge and attitudes among Nigerian women between 2007 and 2017 using multiple Indicator Cluster Survey data. BMC Public Health.

[13] Kareem YO, Dorgbetor CI, Ameyaw EK, Abubakar Z, Adelekan B, Goldson E (2023). Assessment and associated factors of comprehensive HIV knowledge in an at-risk population: a cross-sectional study from 19,286 young persons in Nigeria. Therapeutic Adv Infect Disease.

[14] Badru T, Mwaisaka J, Khamofu H, Agbakwuru C, Adedokun O, Pandey SR (2020). HIV comprehensive knowledge and prevalence among young adolescents in Nigeria: evidence from Akwa Ibom AIDS indicator survey, 2017. BMC Public Health.

[15] UNAIDS. Five questions about the HIV response in Nigeria 2021 [Available from: https://www.unaids.org/en/resources/presscentre/featurestories/2021/october/five-questions-nigeria#:~:text=HIV%20treatment%20coverage%20has%20leapt,no%20risk%20of%20transmitting%20it.

[16] Nazziwa J, Faria NR, Chaplin B, Rawizza H, Kanki P, Dakum P (2020). Characterisation of HIV-1 Molecular Epidemiology in Nigeria: Origin, Diversity, Demography and Geographic Spread. Sci Rep.

[17] Stelzle D, Tanaka LF, Lee KK, Ibrahim Khalil A, Baussano I, Shah ASV (2021). Estimates of the global burden of Cervical cancer associated with HIV. Lancet Glob Health.

[18] Musa J, Achenbach CJ, Evans CT, Jordan N, Daru PH, Hou L (2018). Association between patient-reported HIV status and provider recommendation for screening in an opportunistic Cervical Cancer screening setting in Jos, Nigeria. BMC Health Serv Res.

[19] Croswell JM, Corley DA, Lafata JE, Haas JS, Inadomi JM, Kamineni A (2021). Cancer screening in the U.S. through the COVID-19 pandemic, recovery, and beyond. Prev Med.

[20] Sharpless NE. COVID-19 and cancer. American Association for the Advancement of Science; 2020. p. 1290.

[21] Chenneville T, Gabbidon K, Hanson P, Holyfield C (2020). The impact of COVID-19 on HIV treatment and research: a call to action. Int J Environ Res Public Health.

[22] Merrell K, Ochieng P, Osei-Bonsu E, Seife E, Kemper K, Begna K (2021). 1622P the impact of COVID-19 on cancer treatment delivery in Sub-saharan Africa. Ann Oncol.

[23] Znaor A, Eser S, Anton-Culver H, Fadhil I, Ryzhov A, Silverman BG (2018). Cancer surveillance in northern Africa, and central and western Asia: challenges and strategies in support of developing cancer registries. Lancet Oncol.

[24] Jardim CGR, Zamani R, Akrami M (2022). Evaluating the impact of the COVID-19 pandemic on accessing HIV services in South Africa: a systematic review. Int J Environ Res Public Health.

[25] Adugna A, Azanaw J, Sharew Melaku M (2021). The Effect of COVID-19 on routine HIV Care services from Health Facilities in Northwest Ethiopia. HIV AIDS (Auckl).

[26] Musa J, Achenbach CJ, Evans CT, Jordan N, Daru PH, Hou L (2018). Association between patient-reported HIV status and provider recommendation for screening in an opportunistic Cervical Cancer screening setting in Jos, Nigeria. BMC Health Serv Res.

[27] Dryhurst S, Schneider CR, Kerr J, Freeman AL, Recchia G, Van Der Bles AM (2020). Risk perceptions of COVID-19 around the world. J Risk Res.

[28] Katbi M, Adedoyin A, Meri H, Klindera K, Adegboye A, Abubakar A et al. The impact of covid-19 restrictions on Hiv services among key populations in Nigeria. Top Antiviral Med. 2021:287-.

[29] Hogan AB, Jewell BL, Sherrard-Smith E, Vesga JF, Watson OJ, Whittaker C (2020). Potential impact of the COVID-19 pandemic on HIV, Tuberculosis, and Malaria in low-income and middle-income countries: a modelling study. Lancet Glob Health.

[30] Ford N, Geng E, Ellman T, Orrell C, Ehrenkranz P, Sikazwe I (2020). Emerging priorities for HIV service delivery. PLoS Med.

[31] Spinelli MA, Jones BLH, Gandhi M (2022). COVID-19 outcomes and risk factors among people living with HIV. Curr HIV/AIDS Rep.

[32] Yang X, Sun J, Patel RC, Zhang J, Guo S, Zheng Q (2021). Associations between HIV Infection and clinical spectrum of COVID-19: a population level analysis based on US National COVID Cohort Collaborative (N3C) data. Lancet HIV.

[33] Frescura L, Godfrey-Faussett P, Feizzadeh AA, El-Sadr W, Syarif O, Ghys PD (2022). Achieving the 95 95 95 targets for all: a pathway to ending AIDS. PLoS ONE.

